# Psoriasiform Dermatitis Following JAK Inhibitor Therapy for Hidradenitis Suppurativa and Crohn’s Disease

**DOI:** 10.7759/cureus.114015

**Published:** 2026-08-05

**Authors:** Gerardo S Caussade-Silvestrini, Cristina P Gerena-Maldonado, Rafael E Rodríguez-Rivera, Natalia A Alvarado-Ramos, Julio E Sánchez, Alma Cruz

**Affiliations:** 1 Department of Dermatology, University of Puerto Rico, Medical Sciences Campus, San Juan, USA; 2 Department of Otolaryngology, University of Puerto Rico, Medical Sciences Campus, San Juan, USA

**Keywords:** crohn's disease, hidradenitis suppurativa, jak inhibitor, paradoxical reaction, psoriasiform dermatitis, upadacitinib

## Abstract

Upadacitinib, a selective JAK1 inhibitor approved for hidradenitis suppurativa (HS) and Crohn's disease (CD), has been implicated in paradoxical inflammatory reactions; however, upadacitinib-induced psoriasiform dermatitis in patients treated for HS has not been previously described to the best of our knowledge. We report a 55-year-old woman with HS, CD, rheumatoid arthritis, and systemic lupus erythematosus, receiving upadacitinib 30 mg daily, who developed a pruritic erythematous scaly plaque on the lower back without personal or family history of psoriasis. Punch biopsy demonstrated psoriasiform dermatitis with acanthosis, focal hypogranulosis, uniform elongation of rete ridges, spongiosis, parakeratosis, and a superficial perivascular lymphocytic infiltrate. Given upadacitinib's established efficacy for both HS and CD, the drug was continued, and topical augmented betamethasone dipropionate was initiated, achieving adequate control at two-week follow-up. This case inverts the established paradigm in which JAK inhibitors serve as rescue agents for paradoxical reactions triggered by anti-TNF-α biologics. Comparable psoriasiform eruptions have been reported with baricitinib and tofacitinib, primarily in rheumatoid arthritis, a comorbidity present in our patient and an important interpretive caveat. We hypothesize that interferon-gamma (IFN-γ)-mediated STAT1 dysregulation intrinsic to HS pathogenesis may lower the threshold for JAK inhibitor-induced immune imbalance, although this mechanism remains speculative and warrants further investigation. Paradoxically, upadacitinib has also resolved psoriasiform eruptions in HS, underscoring the bidirectional and unpredictable nature of JAK1 inhibitor-mediated immune modulation. Psoriasiform dermatitis may represent a paradoxical cutaneous reaction to upadacitinib in patients with HS, warranting vigilant dermatologic monitoring and further investigation into individual susceptibility factors.

## Introduction

Hidradenitis suppurativa (HS) is a chronic, debilitating inflammatory skin disease affecting approximately 1% of the general population and characterized by recurrent painful nodules, abscesses, and sinus tract formation predominantly in apocrine gland-bearing skin [1]. Its immunopathogenesis involves a Th1/Th17-dominant inflammatory axis with lesional skin exhibiting elevated expression of IL-17A, IL-17F, and IFN-γ at levels comparable to those observed in psoriasis [1]. Management is guided by disease severity, where topical and systemic antibiotics are used for mild presentations and targeted immunomodulatory therapy is indicated for moderate-to-severe disease. Among emerging options, JAK inhibitors have drawn considerable interest for their ability to simultaneously modulate multiple cytokine signaling pathways implicated in HS pathogenesis [2]. Upadacitinib, a second-generation selective JAK1 inhibitor, selectively inhibits JAK1-mediated signaling of proinflammatory cytokines, including interleukin (IL)-6 and interferon-gamma (IFN-γ) [3]. It has demonstrated significant efficacy in moderate-to-severe HS and is approved for several overlapping immune-mediated diseases, including Crohn's disease (CD), making it an attractive therapeutic option for patients with multiple inflammatory comorbidities [2,3].

Despite their therapeutic benefits, targeted immunotherapies, including JAK inhibitors, can be associated with paradoxical inflammatory cutaneous reactions. This is defined as the new onset or exacerbation of an immune-mediated condition during treatment of another [4]. Current literature has described such reactions with a range of biologics, including anti-TNF agents, IL-17 inhibitors, and IL-4/IL-13 blockers, and they are now increasingly recognized with JAK inhibitors as well [4,5]. Psoriasiform dermatitis is one example: a group of inflammatory skin disorders sharing clinical and histological features with psoriasis of erythematous scaling plaques, epidermal hyperplasia, parakeratosis, and superficial perivascular lymphocytic infiltrate yet lacking the complete molecular signature of classical psoriasis and demonstrating more variable activation of inflammatory pathways depending on the underlying trigger [5,6].

To date, JAK inhibitor-associated psoriasiform eruptions have been described mainly in the context of rheumatoid arthritis (RA) and atopic dermatitis. These eruptions have been hypothesized to result from a disruption of the homeostatic balance between IFN-γ and IFN-α following JAK pathway downregulation [5]. Given the limited number of published JAK inhibitor-associated psoriasiform eruptions to date, whether this phenomenon can arise in patients treated with JAK inhibitors specifically for HS remains understudied, reflecting an important gap in the current literature. Here, we report a case of psoriasiform dermatitis developing in a patient receiving upadacitinib for HS and CD and discuss the implications for cutaneous monitoring in this growing patient population.

## Case presentation

**Figure 1:**
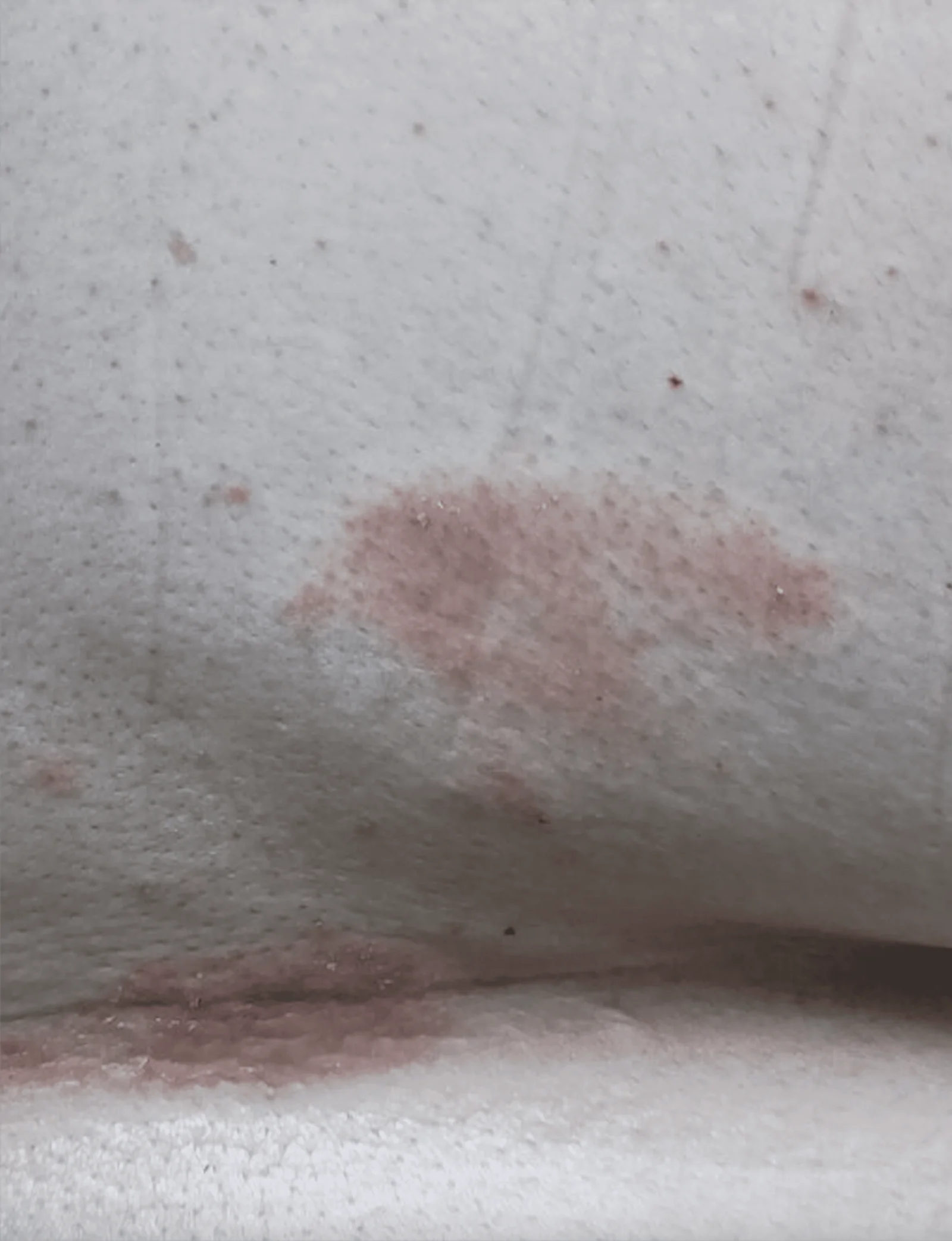
Clinical photograph showing erythematous, scaly plaques on the lower back, consistent with psoriasiform dermatitis.

A 55-year-old woman with a past medical history significant for HS, CD, RA, and systemic lupus erythematosus (SLE), currently on upadacitinib 30 mg orally once daily, presented at our HS specialized dermatology clinic with a pruritic back lesion of approximately four months’ duration. Her primary care physician had prescribed topical acyclovir due to suspicion of shingles, with no improvement. On examination, the lesion appeared as erythematous scaly plaques on the lower back (Figure 1). No active HS lesions were noted at that visit, indicating that her HS remained well controlled while receiving upadacitinib. She had no personal or family history of psoriasis.

Four years prior, at age 51, the patient had first been evaluated at our clinic with a physical examination of bilateral axillary and inframammary scarring with comedones, a buttock inflammatory nodule, and a perianal fistula, consistent with a diagnosis of HS Hurley Stage 2. Her CD was also active at that time, prompting a multidisciplinary treatment approach with gastroenterology. Infliximab was initiated to manage HS and CD simultaneously, resulting in clinical improvement in both conditions. Approximately two years later, infliximab was discontinued due to the development of acute panniculitis, and the patient was transitioned to upadacitinib 30 mg orally once daily, achieving excellent control of both conditions. She had been receiving upadacitinib for approximately seven months prior to the onset of the cutaneous eruption.

**Figure 2:**
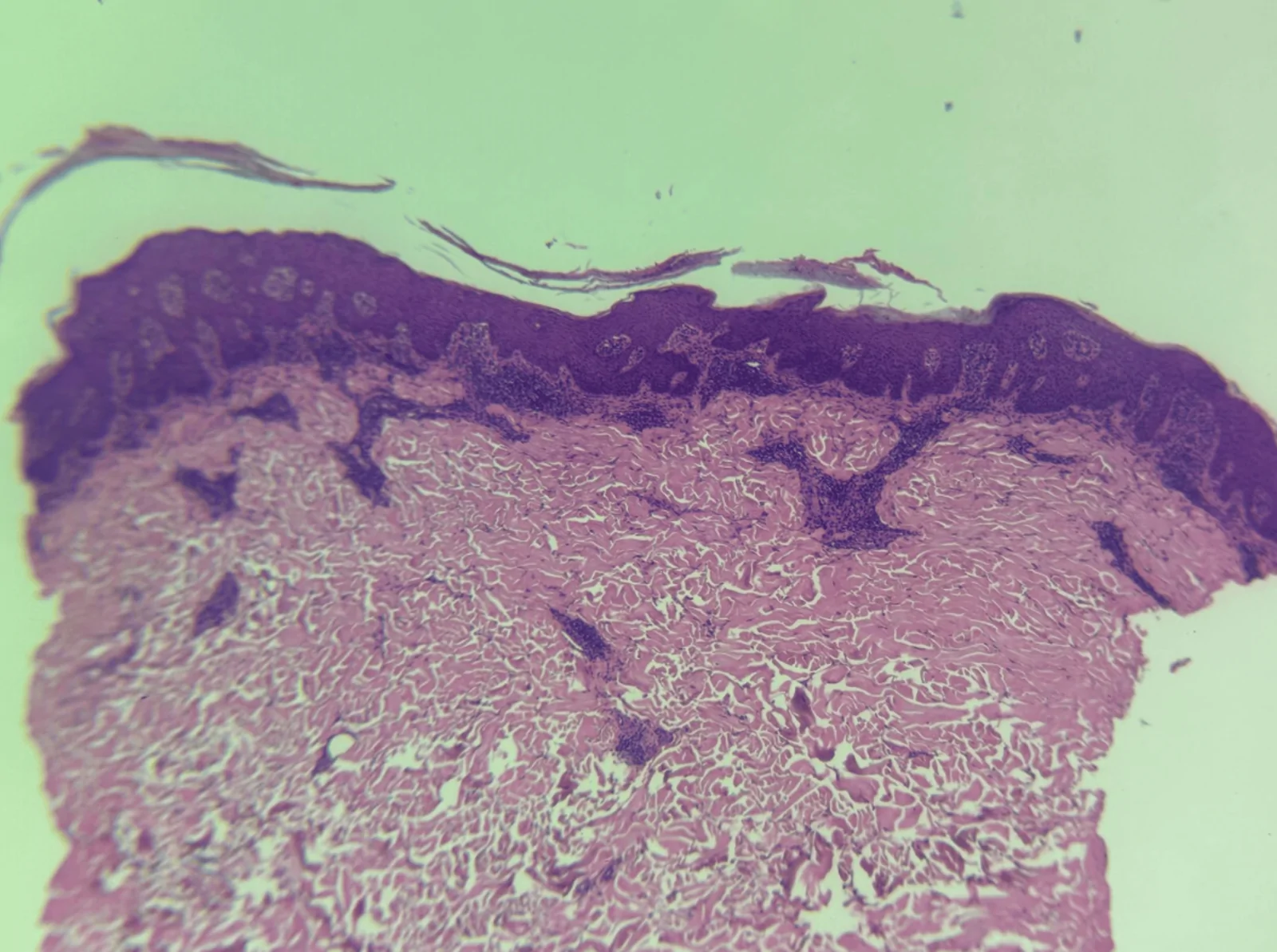
Low-power view (4×) demonstrating epidermal hyperplasia with a psoriasiform pattern and overlying compact stratum corneum with focal serum crust; a superficial dermal inflammatory infiltrate is present.

**Figure 3:**
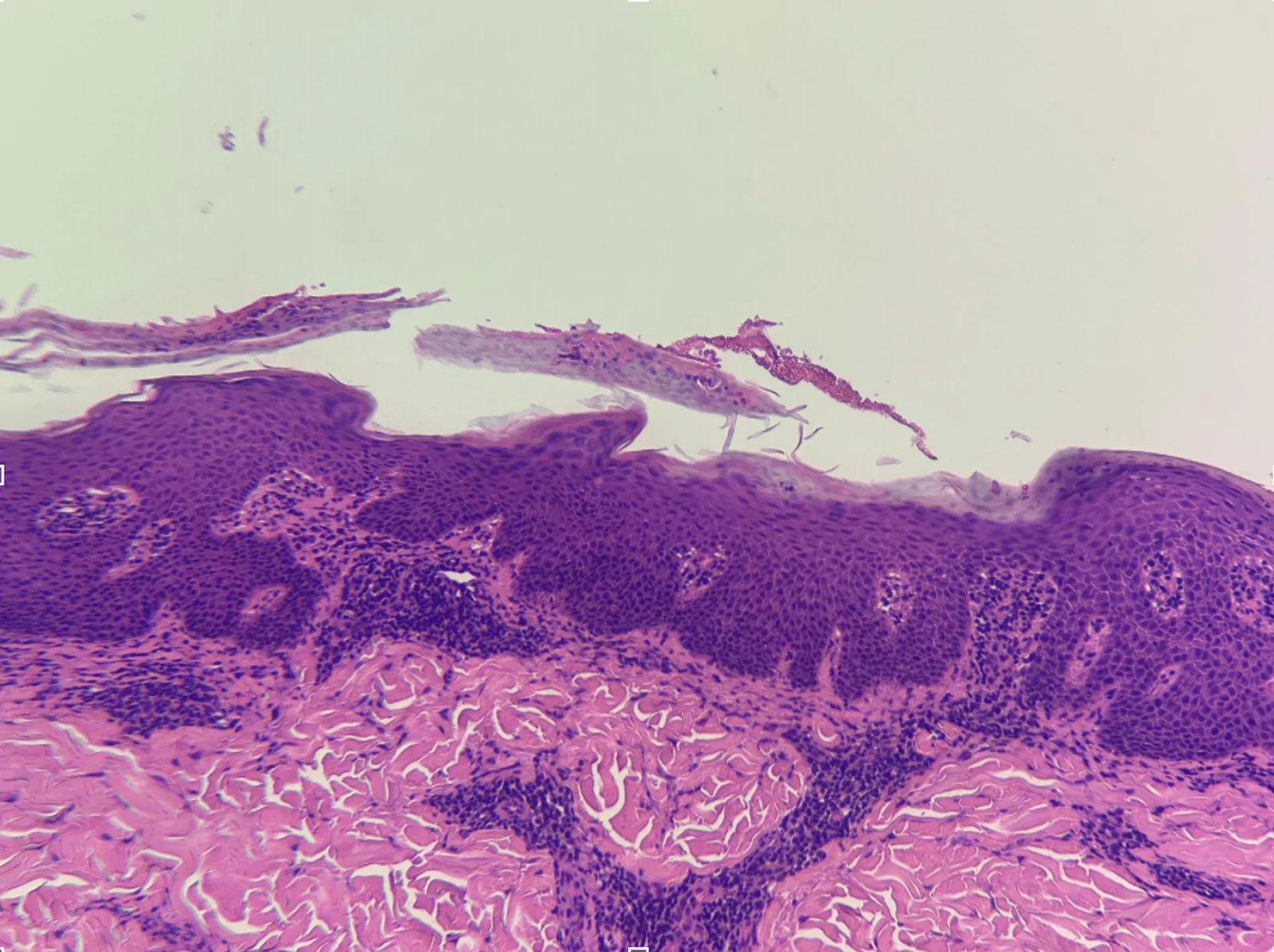
Intermediate-power view (10×) demonstrating acanthotic epidermis with psoriasiform hyperplasia and uniform elongation of rete ridges, accompanied by focal hypogranulosis, mild spongiosis, and a superficial dermal inflammatory infiltrate.

**Figure 4:**
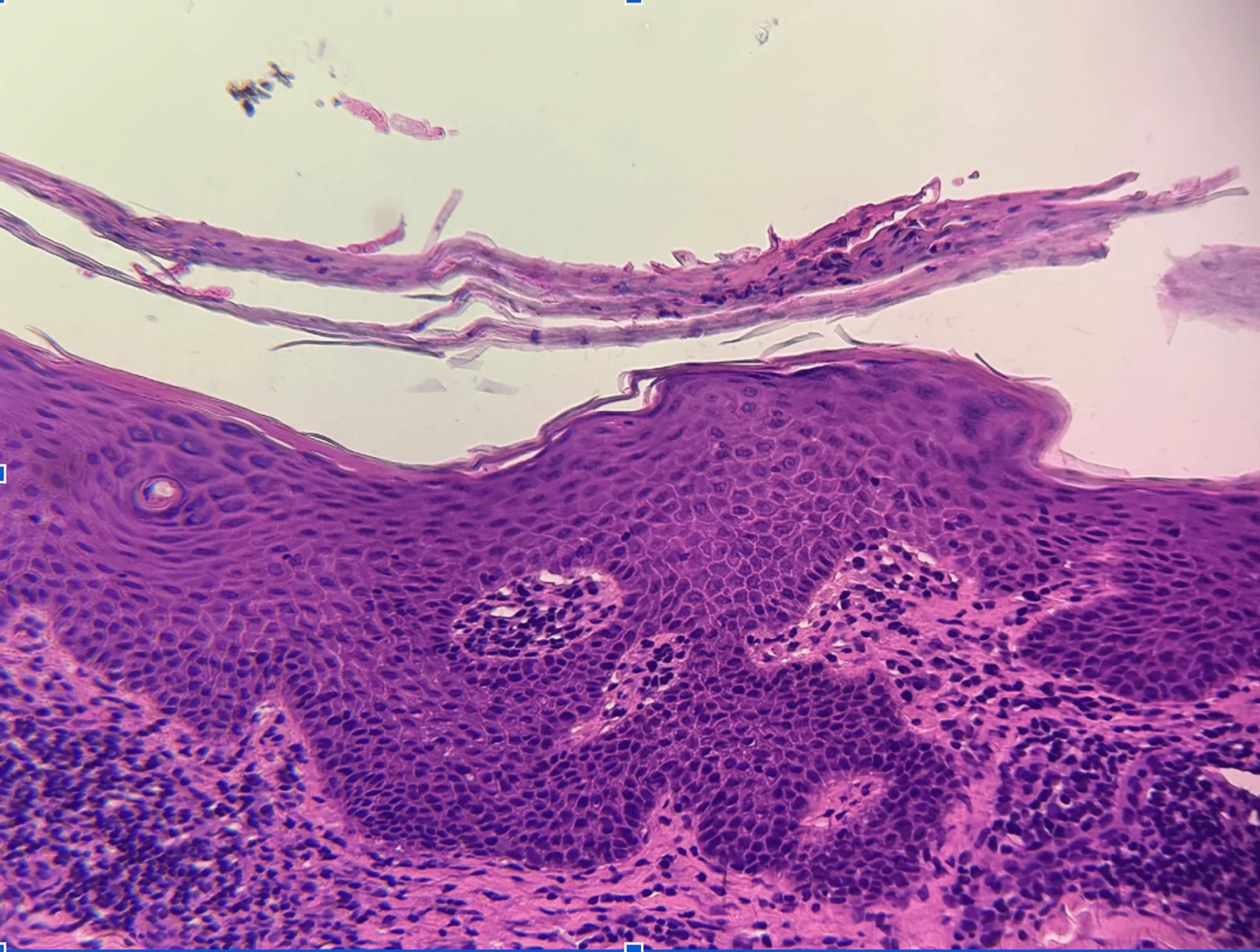
Higher-power view (20×) demonstrating acanthotic epidermis with psoriasiform hyperplasia and uniform elongation of rete ridges, with focal spongiosis and an overlying detached fragment of stratum corneum.

**Figure 5:**
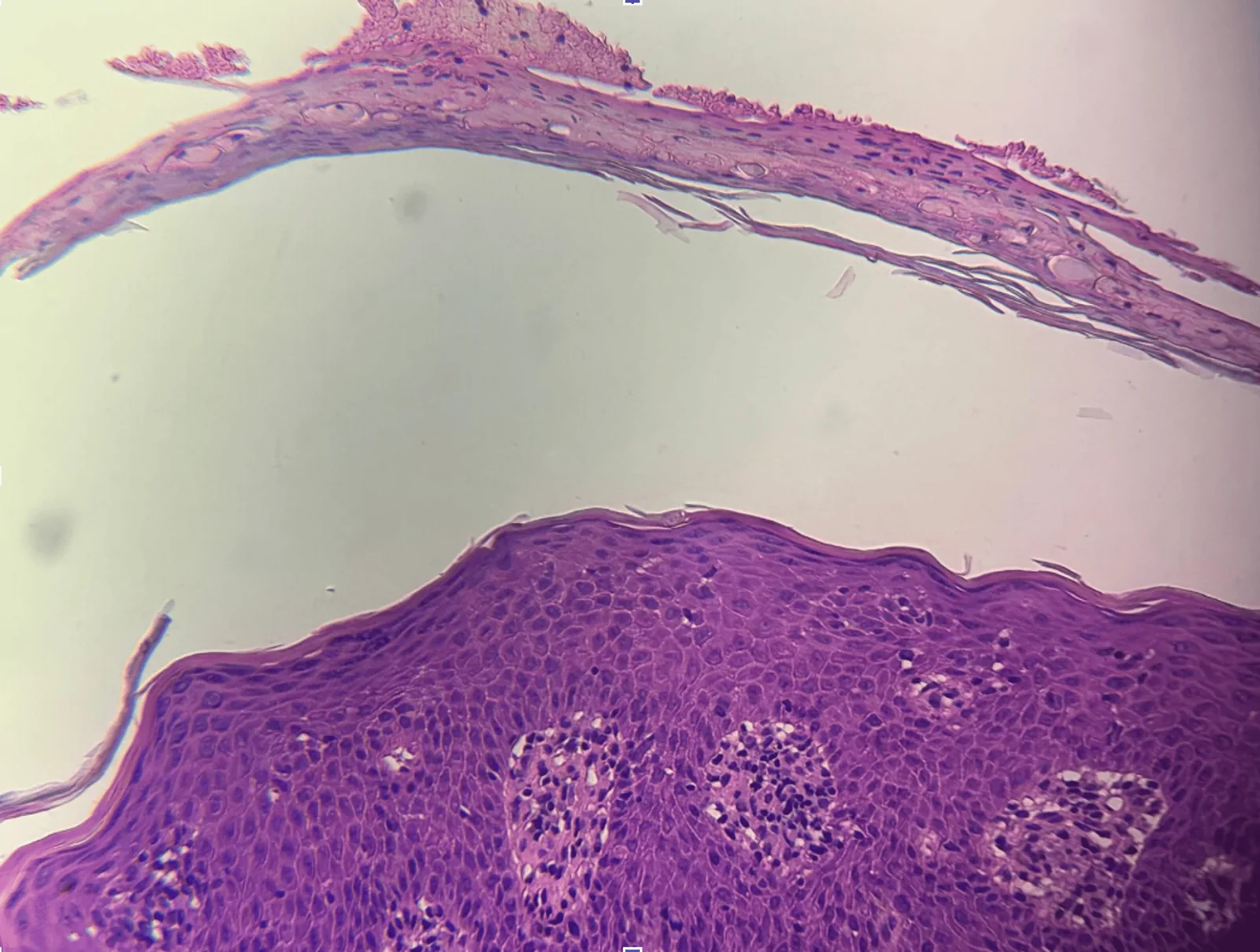
Higher-power view (20×) demonstrating a detached fragment of stratum corneum with serum deposition and overlying crust, above an acanthotic epidermis with psoriasiform hyperplasia.

A 4 mm punch biopsy of the back lesion was performed at this visit, revealing an acanthotic epidermis with focal areas of hypogranulosis, uniform elongation of rete ridges in a psoriasiform pattern, spongiosis, parakeratosis, a serum deposit with overlying crust within the stratum corneum, and a superficial perivascular lymphocytic infiltrate, consistent with psoriasiform dermatitis (Figures 2-5). The differential diagnosis included eruptive psoriasis and nummular dermatitis. Tinea was also considered given its potential to clinically mimic psoriasiform dermatitis; however, no fungal hyphae were identified on routine histopathologic examination, and the clinical presentation and course were not consistent with a dermatophyte infection. Although a dedicated potassium hydroxide (KOH) preparation or Periodic acid-Schiff (PAS) stain was not performed, the overall clinicopathologic findings made a fungal etiology unlikely.

Considering the temporal relationship between upadacitinib initiation and rash onset, previously published reports of similar reactions, and objective clinical and histopathologic findings, while acknowledging the absence of drug rechallenge and the presence of potential confounding autoimmune comorbidities, the Naranjo Adverse Drug Reaction Probability Scale was applied. It yielded a score of +3, consistent with a possible adverse drug reaction. Nevertheless, given the established efficacy of upadacitinib in controlling her HS and CD, the decision was made to continue the medication and to manage the cutaneous eruption with topical augmented betamethasone dipropionate ointment twice daily as needed. At the two-week follow-up, the psoriasiform dermatitis remained adequately controlled with topical therapy, without dose modification of upadacitinib.

## Discussion

Drug-induced eruptions represent an important and increasingly recognized trigger for psoriasiform dermatitis, with growing evidence implicating targeted immunotherapies, including anti-TNF agents, IL-17 inhibitors, IL-4/IL-13 blockers, and JAK inhibitors, in their pathogenesis [5,7,8]. Despite sharing clinical and histological features with classical psoriasis, drug-induced psoriasiform dermatitis lacks its full molecular signature, underscoring the need to identify triggers to guide management.

Psoriasis is driven by a Th1 and Th17 inflammatory axis, in which key cytokines such as IL-17, IL-23, and tumor necrosis factor-α (TNF-α) are involved in keratinocyte proliferation and sustained inflammation [5,9]. In contrast, psoriasiform dermatitis demonstrates a more variable and incomplete activation of these pathways, which depends significantly on the underlying trigger [5,6]. Meanwhile, HS shares a similar immunologic profile with psoriasis, where both are characterized by a Th1/Th17-dominant axis. Specifically, HS lesions exhibit an elevated expression of Th1 cytokine IFN-γ, as well as Th17-associated cytokines IL-17A and IL-17F [5,9].

Anti-TNF agents remain an established therapeutic option for patients with concomitant HS and CD because of their efficacy in both conditions [10]. In our patient, however, infliximab was discontinued following the development of acute panniculitis despite achieving satisfactory control of both diseases. Consequently, upadacitinib was selected as an alternative therapy to maintain disease control, providing important clinical context for interpreting the subsequent development of the psoriasiform eruption.

In our patient, upadacitinib 30 mg daily achieved excellent control of both her HS and CD yet was associated with the development of a psoriasiform skin eruption, consistent with paradoxical reactions reported with other JAK inhibitors. The suspected paradoxical reaction observed in our patient has been reported with other JAK inhibitors, primarily in the context of RA [5]. In one case, a patient developed a psoriasiform eruption on the scalp and upper limbs three weeks after beginning baricitinib [5]. In another similar case, eruptions appeared on the arms, legs, and ankles three months after starting tofacitinib [5]. Like our patient, neither had a personal or family history of psoriasis. Notably, our patient also had a diagnosis of RA, the same condition in which these prior reactions were reported, which introduces an important interpretive caveat. It is possible that RA-related immune dysregulation, rather than HS alone, contributed to her susceptibility to this paradoxical eruption. This shared comorbidity should be considered when evaluating the HS-specific nature of this association, and further cases of HS without RA will be necessary to establish a clearer causal link. Nevertheless, there is a mechanistic rationale for an HS-specific susceptibility: IFNγ-mediated STAT1 activation has been implicated in the pathogenesis of HS [9], suggesting that patients with HS may carry a pre-existing dysregulation of IFN signaling that could amplify the immune imbalance induced by JAK inhibition and thereby lower the threshold for paradoxical cutaneous reactions. The proposed pathogenesis involves immune pathway dysfunction in which JAK downregulation disrupts the homeostatic balance between IFN-γ and IFN-α [5], though the full scope of these mechanisms, particularly as they interact with the inflammatory milieu of HS, remains to be defined. 

Although the Naranjo Adverse Drug Reaction Probability Scale supported a possible association between upadacitinib and the psoriasiform eruption in this patient, the coexistence of multiple autoimmune diseases and the absence of drug rechallenge preclude definitive causal inference [11]. Additional studies are needed to define the immunologic mechanisms underlying JAK inhibitor-induced paradoxical psoriasiform dermatitis and to determine whether autoimmune comorbidities such as HS, RA, CD, or SLE modify susceptibility to this reaction.

## Conclusions

Overall, upadacitinib and other JAK inhibitors offer significant therapeutic benefits across multiple inflammatory conditions but may, in rare cases, be associated with paradoxical cutaneous reactions such as the psoriasiform dermatitis observed in this patient, which we interpret as a possible paradoxical reaction rather than an established causal effect. Vigilant clinical monitoring is essential to recognize these manifestations early and manage them appropriately, particularly in patients with complex multimorbidity. The conclusions drawn from this report are inherently limited by its single-patient design. Broader case series and rigorously designed observational studies are needed to confirm whether this association is genuine, elucidate its underlying immunopathogenic mechanisms, identify predisposing factors, and determine its reproducibility across diverse patient populations.
